# Deficiency in RCAT-1 Function Causes Dopamine Metabolism Related Behavioral Disorders in *Caenorhabditis elegans*

**DOI:** 10.3390/ijms23042393

**Published:** 2022-02-21

**Authors:** Haelim Jeong, Jun Young Park, Ji-Hyun Lee, Ja-Hyun Baik, Chae-Yeon Kim, Jin-Young Cho, Monica Driscoll, Young-Ki Paik

**Affiliations:** 1 Department of Biochemistry, College of Life Sciences and Biotechnology, Yonsei University, Seoul 03722, Korea; jeongh85@gmail.com (H.J.); jihyun_lee@cj.net (J.-H.L.); 2 Yonsei Proteome Research Center, Yonsei University, Seoul 03722, Korea; parkjy@proteomix.org (J.Y.P.); kimcy@proteomix.org (C.-Y.K.); chojy@proteomix.org (J.-Y.C.); 3 Department of Life Sciences, Korea University, Seoul 02841, Korea; jahyunb@korea.ac.kr; 4 Interdisciplinary Program in Integrative Omics for Biomedical Science, Yonsei University, Seoul 03722, Korea; 5 Department of Molecular Biology and Biochemistry, Rutgers University, Piscataway, NJ 08855, USA; driscoll@biology.rutgers.edu

**Keywords:** *C. elegans*, dopamine, dopamine dysregulation syndrome, Parkinson’s disease, vesicular monoamine transporter, tyrosine hydroxylase

## Abstract

When animals are faced with food depletion, food search-associated locomotion is crucial for their survival. Although food search-associated locomotion is known to be regulated by dopamine, it has yet to investigate the potential molecular mechanisms governing the regulation of genes involved in dopamine metabolism (e.g., *cat-1, cat-2*) and related behavioral disorders. During the studies of the pheromone ascaroside, a signal of starvation stress in *C. elegans,* we identified R02D3.7, renamed *rcat-1* (regulator of *cat* genes-*1*), which had previously been shown to bind to regulatory sequences of both *cat-1* and *cat-2* genes. It was found that RCAT-1 (R02D3.7) is expressed in dopaminergic neurons and functions as a novel negative transcriptional regulator for *cat-1* and *cat-2* genes. When a food source becomes depleted, the null mutant, *rcat-1(ok1745)*, exhibited an increased frequency of high-angled turns and intensified area restricted search behavior compared to the wild-type animals. Moreover, *rcat-1(ok1745)* also showed defects in state-dependent olfactory adaptation and basal slowing response, suggesting that the mutants are deficient in either sensing food or locomotion toward food. However, *rcat-1(ok1745)* has normal cuticular structures and locomotion genes. The discovery of *rcat-1* not only identifies a new subtype of dopamine-related behaviors but also provides a potential therapeutic target in Parkinson’s disease.

## 1. Introduction

Environmental stresses, such as food shortage, temperature changes and an increase in population density, largely influence the adaptation and survival of animals. When animals are exposed to food depletion, food search-associated locomotion is crucial for their survival and evolution. For example, the nematode *Caenorhabditis elegans* actively migrates toward food-abundant areas or starts searching for food to support its survival, development, and reproduction. *C. elegans* has developed strategies to avoid risks caused by basal slowing response (BSR) when it encounters food and to increase the time spent with the food source [1]. When a food source becomes depleted, the worms tend to increase their locomotion rate so that they find more food. Furthermore, *C. elegans* makes an area-restricted search (ARS) using frequent high-angled turning as it attempts to find food resources in environments lacking food [2].

Biogenic amines (e.g., dopamine, serotonin) are known to play important roles in such food search behavior in *C. elegans* [3]. In particular, dopamine regulates BSR, ARS and egg-laying behavior in *C. elegans* [1,2,4]. The overall activity level of dopamine neurons appears to produce parallel changes in motor activity in *C. elegans* and mammals [5]. Recently, it was reported that supply of exogenous dopamine restores moving activity and improves body length defects caused by simulated microgravity [6]. Moreover, normal dopamine function appears to be required for the establishment and maintenance of incentive learning in animals [5,7]. Dopamine is also required for the generation of state-dependency, a learned behavior in *C. elegans* in which the animals recall their environment and modify their olfactory adaptation in the presence of a neuroactive drug [8]. Because dopamine signaling is associated with motor control, memory and adaptation in both invertebrates and vertebrates [7], *C. elegans* is a suitable model animal to study dopamine-related diseases, such as Parkinson’s disease (PD) [9].

During studies of the transcriptional regulation of genes involved in signal transduction in response to the pheromone ascaroside, which usually serves as a signal of food depletion or environmental stress caused by population density in *C. elegans* [10,11], we identified the *R02D3.7* gene as a candidate modulator of dopamine-related behaviors during food searching and found evidence for its potential function in the dopaminergic signaling pathway. Here, we show that R02D3.7 is a novel negative transcriptional regulator of both *cat-1* and *cat-2*. *cat-1* is the homolog of the mammalian vesicular monoamine transporter VMAT2, and it plays a key role in dopamine release and packaging in *C. elegans* [12]. *cat-2* is the homolog of mammalian tyrosine hydroxylase *(TH)* and is involved in dopamine biosynthesis in *C. elegans* [13]. Given that *R02D3.7* was previously shown to bind to the promoters of both *cat-1* and *cat-2*, we renamed *R02D3.7* as *rcat-1* (regulator of cat genes). Furthermore, we propose that a null mutant strain of this gene may be used to generate an in vivo model animal to study dopamine dysregulation syndrome (DDS) in PD patients, because it results in behavioral defects that are related to DDS and other dopamine-related disorders (e.g., dopamine addiction).

## 2. Results

R02D3.7 was identified during our early work on ascaroside signaling [10,11]. To explore biological functions of *R02D3.7,* which was predicted to contain two zinc-finger binding motifs that bind 500 bp upstream of the *daf-7* gene [14], we attempted to characterize the role of R02D3.7 by examining noticeable differences in behaviors in *R02D3.7* mutants that had been backcrossed six times with N2 wild-type animals. Using multiple behavioral assays including ARS and state-dependency olfactory adaptation, we compared the behaviors of *R02D3.7* mutants to those of wild-type animals. After determining the *R02D3.7* mutant defect in multiple dopamine related behaviors, we assessed *R02D3.7* localization by GFP-tagging and we monitored transcription of dopamine metabolism genes in *R02D3.7* mutants.

### 2.1. Behavioral Assays Using R02D3.7(ok1745)

#### 2.1.1. Turning Frequency of *R02D3.7(ok1745)*

**Figure 1:**
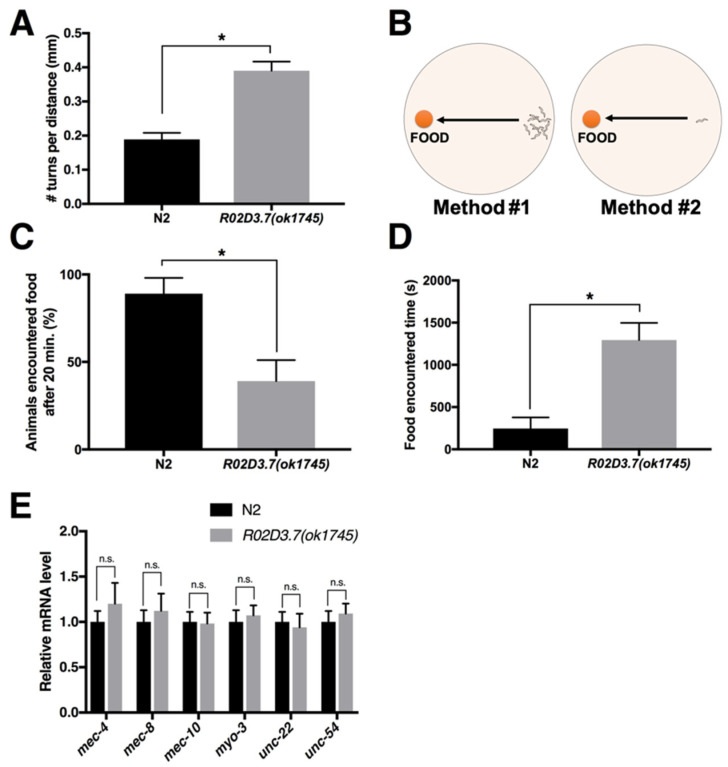
*R02D3.7(ok1745)* mutants exhibit defects in turning frequency and food-response locomotion. (**A**) The turning frequency of N2 wild-type and *R02D3.7(ok1745)* mutants. Bars represent the mean ± standard deviation (s.d.); * *p* < 0.0001; two-tailed, unpaired Student’s *t*-test; *n =* 124 for N2; *n =* 143 for *R02D3.7(ok1745)*. (**B**) Schematic of food-encountering assay. Method #1 was used to determine the number of animals that encountered food in a 20-min period, whereas method #2 was used to measure the time an animal took to reach the food. (**C**) Relative ratio of wildtype and *R02D3.7(ok1745)* mutants encountering food in a 20-min period (method #1). Bars represent the mean ± s.d.; * *p* < 0.0001; two-tailed, unpaired Student’s *t*-test. Three independent experiments were performed with 120 worms of each strain. (**D**) Measuring the time taken to reach food in wild-type and *R02D3.7(ok1754)* animals. The bars represent the mean ± s.d.; * *p* < 0.0001; two-tailed, unpaired Student’s *t*-test; *n =* 144 for N2; *n =* 155 for *R02D3.7(ok1754)*. (**E**) Mechanosensation and movement related gene expression in N2 and *R02D3.7*(*ok1745)* strains. n.s., no significant difference (*p* > 0.5); significance was determined using a two-tailed, unpaired *t*-test. Bars are mean ± s.d. of three independent biological replicates.

We checked the initial response to food of the R02D3.7(*ok1745*) mutants. We found that once *R02D3.7(ok1745)* mutants are removed from food or moved to a no-food area, mutant worms had difficulties in food search-associated locomotion (Videos S1 and S2). That is, whereas wild-type animals traveled actively to find food, *R02D3.7(ok1745)* animals remained in a food-absent area without any noticeable efforts to search for food. To determine the physiological basis of such phenotypic changes (i.e., locomotion deficiency for food searching) in *R02D3.7* mutants, we then measured their turning frequency. We compared the number of turns made within the traveled distance and found that *R02D3.7* mutants made more turns than wild-type N2 animals over the same distance (Figure 1A), indicating that the mutants moved a much shorter distance in a given time period than the wild-type N2 animals, as turning frequency is inversely proportional to the distance traveled. When we carefully tracked the patterns of egg deposition relative to food, we noted that *R02D3.7* mutants laid eggs outside the seeded food area (Figure S1), in contrast to N2 wild-type animals, which usually laid eggs near food (seeded *Escherichia coli*) [15,16]. This indicated that the *R02D3.7*(*ok1745*) mutants may have a defect in either sensing food or locomotion toward food.

#### 2.1.2. Food Detection Assay of *R02D3.7(ok1745)*

In order to quantify the food-search behavior of *R02D3.7* mutants, we performed a chemotaxis assay (Figure 1B) [17]. First, we measured the percentage of the population that moved toward food. Then, we measured the time taken for one worm to reach the food. When the first method was used, only approximately 40% of the *R02D3.7* mutants, relative to the number of wild-type animals, reached the food in a given 20-min period (Figure 1C). This result indicated that *R02D3.7(ok1745)* animals may have defects in locomotion toward food. In complementary tests that measured individual animals’ food-search speed, *R02D3.7(ok1745)* mutants took an average of 1300 s to reach the food, whereas N2 animals took only approximately 250 s, which was >5-fold faster than the mutants (Figure 1D).

#### 2.1.3. *R02D3.7(ok1745)* Shows No Defects in Cuticular Structures

We used DiI staining of the cuticular structures of chemosensory neurons to determine whether behavioral defects of *R02D3.7(ok1745)* are attributable to the abnormal structures of chemosensory neurons in the mutant background [18]. There were no discernable differences in the pattern of stained cuticular structures between wild-type and *R02D3.7(ok1745)* mutants (Figure S2). Moreover, the mRNA levels of genes involved in locomotion regulation, such as *mec-4* (amiloride-sensitive Na+ channel), *mec-8* (required for body wall muscle development), *mec-10* (amiloride-sensitive Na+ channel), *myo-3* (myosin heavy chain), *unc-22* (twitchin) and *unc-54* (twitchin) [19,20], remained unchanged in *R02D3.7(ok1745)* mutants (Figure 1E). The lack of detectable changes in structure of chemosensory neurons that participate in food searching behavior [18] or in locomotion gene expression suggest that the *R02D3.7(ok1745)* mutant deficits may instead be related to the monoaminergic neuronal circuit, which is involved in food searching [2,21].

### 2.2. R02D3.7 Is Expressed in Dopaminergic Neurons

**Figure 2:**
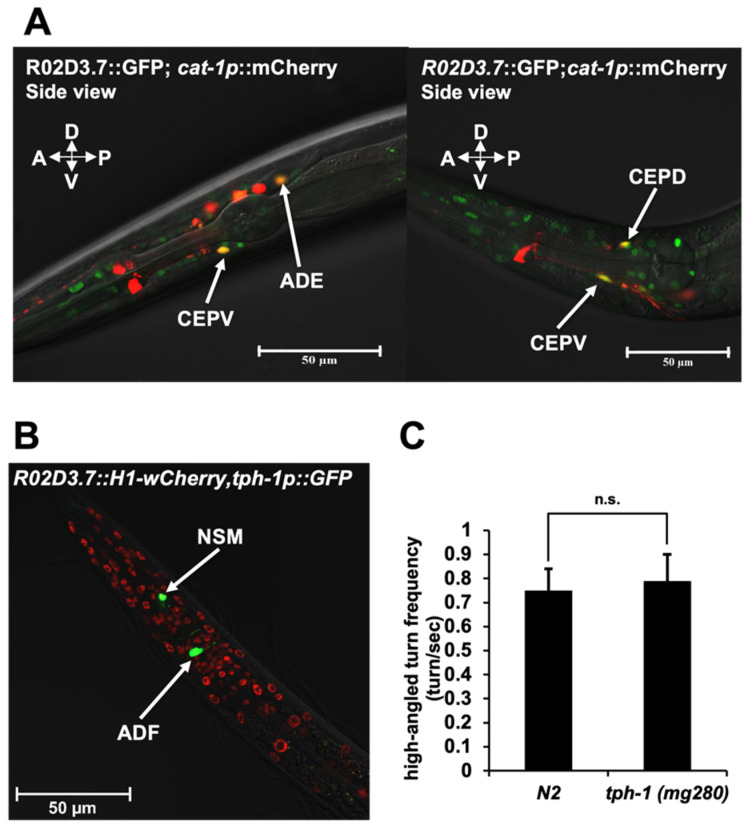
R02D3.7 expression in dopaminergic neurons. (**A**) Confocal images of the head regions of *R02D3.7::GFP*; *cat-1p::mCherry* transgenic worms. Each image was captured using a 63× oil-immersion lens on a ZEISS LSM700 confocal microscope. Green indicates *R02D3.7::GFP* expression, red indicates *cat-1p::mCherry* expression and yellow with arrows indicates merged regions. Scale bar = 50 μm. Anterior deirid sensillum (ADE), dorsal cephalic sensillum (CEPD) and ventral cephalic (CEPV) neurons are shown. (**B**) Confocal image of the head regions of *tph-1p::GFP*; *R02D3.7::H1-wCherry* transgenic worms. Images were captured using 40× oil-immersion lens on a ZEISS LSM900 confocal microscope. Green arrows indicate *tph-1p::GFP* expression, and red arrows indicate *R02D3.7::H1-wCherry* expression. Scale bar = 50 μm. ADF and NSM neurons are shown. (**C**) Measurement of high-angled turning frequency in N2 wild-type and *tph-1(mg280)* animals. Bars represent the mean ± s.d.; n.s., no significant difference (*p* > 0.5); significance was determined using a two-tailed, unpaired Student’s *t*-test; three independent biological tests were performed with *n* = 30 for each set.

The *R02D3.7(ok1745)* mutant deficit in ability to reach food-abundant areas led us to hypothesize that this strain may have a deficiency in monoamine metabolism (e.g., dopamine, serotonin), which is known to be involved in the regulation of appetite and food-search behaviors in animals, including *C. elegans* [22]. In a previous study, we found R02D3.7 to be expressed within many tissues, including head neurons [23]. To determine whether R02D3.7 is expressed in monoamine-specific neurons, we microinjected a GFP-tagged R02D3.7 transgenic construct into strain OH9279. This construct included an integrated fluorescent marker for dopaminergic and serotonergic neurons, *cat-1p*::*mCherry* [24]. Using this transgenic line, we found that the expression of the *R02D3.7* translation overlapped with the expression of *cat-1* in dopaminergic neurons (Figure 2A).

*tph-1* encodes tryptophan hydroxylase, which is essential for serotonin biosynthesis and the *tph-1* promoter can drive serotonergic neuron-specific marker expression [25]. Since the *tph-1* mutant is known to exhibit abnormality in food sensation and ingestion in *C. elegans*, we sought to confirm that R02D3.7 is not expressed in serotonergic neurons. To this end, we constructed transgenic lines that co-expressed *R02D3.7::mCherry* and *tph-1::GFP* from their native promoters and examined overlap [26,27]. Our analysis confirmed that *R02D3.7* and *tph-1* are expressed in different neuronal cells (Figure 2B). Furthermore, we observed that serotonin was not required for food-search behavior (Figure 2C). We conclude that R02D3.7 is expressed in dopaminergic neurons, but not serotonergic neurons.

### 2.3. Potential Mechanisms Underlying Excessive Dopamine Production and Release in R02D3.7(ok1745) Mutants

#### 2.3.1. Area Restricted Search (ARS) Behavior of *R02D3.7(ok1745)* Mutants

**Figure 3:**
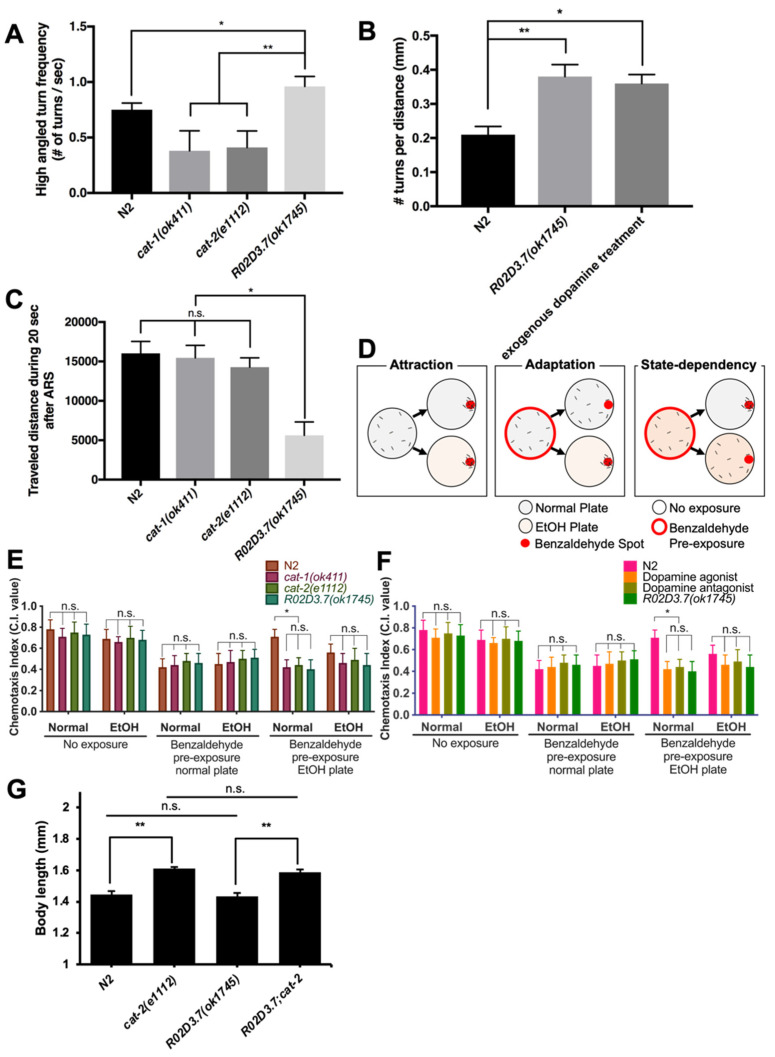
Examination of dopamine-related behavior in *R02D3.7*(*ok1745*) animals. (**A**) ARS behavior assay. Bars represent the mean ± s.d.; * *p* < 0.0005; ** *p* < 0.0001; ordinary one-way analysis of variance (ANOVA), multiple comparisons; *n* = 124 for N2; *n* = 130 for *cat-1(ok411); n* = 114 for *cat-2(e1112)* and *n* = 133 for *R02D3.7(ok1745)*. (**B**) Turning frequency of N2 wild-type and *R02D3.7(ok1745)* mutant animals treated with 1 μM exogenous dopamine. Bars represent mean ± s.d.; * *p* < 0.001; two-tailed, unpaired Student’s *t*-test; three independent biological tests were performed with *n* = 30 for each set. (**C**) Measurement of transit distance in 20 s after ARS under conditions of food scarcity. Bars represent the mean ± s.d.; n.s., no significant difference (*p* > 0.5); * *p* < 0.0001; ordinary one-way ANOVA, multiple comparisons; *n* = 124 for N2; *n* = 130 for *cat-1(ok411); n* = 114 for *cat-2(e1112)* and *n* = 133 for *R02D3.7(ok1745)*. (**D**) A scheme of state-dependent olfactory adaptation assay. Red circular spot indicates the location of benzaldehyde drop and white circle indicates a spot is the initial spot of animals. (**E**) State-dependent olfactory adaptation assay for wild-type, *R02D3.7(ok1745),* and dopamine deficient strains including *cat-1(ok411)* and *cat-2(e1112).* Bars are mean ± s.d.; * *p* > 0.0001; Ordinary one-way ANOVA, multiple comparisons; Seven independent experiments were performed with 100 worms for each strain. (**F**) State-dependent olfactory adaptation assay in presence of exogenous dopamine and exogenous raclopride in wild-type animals. Bars are mean ± s.d.; * *p* > 0.0001; Ordinary one-way ANOVA, multiple comparisons; Seven independent experiments were performed with 100 worms for each strain. (**G**) Epistatic assay for measuring body length. Bars represent the mean ± standard error of the mean; ** *p* < 0.0001; n.s., no significant difference (*p* > 0.5); two-tailed, unpaired Student’s *t*-test; *n* =81 for N2; *n* = 56 for *cat-2(e1112); n* = 55 for *R02D3.7(ok1745)* and *n* = 63 for *R02D3.7;cat-2*.

Based on the results described above (see Figure 1A), we hypothesized that the function of the *R02D3.7* gene may be linked to dopamine neuromodulators that are involved in one of the behaviors of ARS [2]. To test this hypothesis, we first examined whether *R02D3.7(ok1745)* mutants exhibit any deficiency in ARS, which is one of the foraging strategies of *C. elegans* [2], and we found that *R02D3.7(ok1745)* mutants exhibited a notable increase in the number of high-angled turns on a food-containing plate compared with the wild-type animals (Figure 3A). This phenotype differed from the phenotypes of the well-known dopamine metabolism-deficient mutants *cat-1(ok411)* and *cat-2(e1112)* (Figure 3A), which usually exhibit a lower turning frequency than N2 wild-type animals [7]. *cat-1(ok411)* and *cat-2(e1112)* are null mutants for the corresponding gene, like their other mutant alleles, *cat-1(e1111)* and *cat-2(n4547)* [12,28,29,30]. Instead, the phenotype of R02D3.7(*ok1745)* mutants was similar to the phenotype seen after treatment with exogenous dopamine, which results in more frequent turning behavior (Figure 3B) [2]. The intensified ARS behavior in *R02D3.7(ok1745)* mutants appeared to interfere with the dispersion search (Figure 3C). When worms are unable to find food in their local area due to ARS behavior, they use dispersion search behavior to find food in a distant area [2,31]. Notably, *R02D3.7(ok1745)* mutants did not travel as far as the wild-type animals or the dopamine-metabolism-deficient mutants *cat-1(ok411*) and *cat-2(e1112)*) (Figure 3C). Thus, we tentatively concluded that *R02D3.7(ok1745)* mutants exhibited intensified ARS behavior, in that they were unable to perform dispersion search behavior. This finding implies a newly identified subtype of dopamine-related behaviors.

#### 2.3.2. State-Dependency Olfactory Adaptation of *R02D3.7(ok1745)*

State-dependency describes an adaptive response that allows *C. elegans* to disregard an uninformative odorant or modulate its olfactory responses based on experience [8]. We investigated whether *R02D3.7(ok1745)* mutants showed state-dependent olfactory adaptation (Figure 3D). Both *R02D3.7(ok1745)* and dopamine metabolism-deficient mutants *cat-1* and *cat-2* exhibited good sensitivity to ethanol and showed normal chemotaxis and adaptation to benzaldehyde, an attractant for *C. elegans* (Figure 3E) [32]. However, these mutants demonstrated a deficiency in state-dependency in studies in which we pre-exposed animals to benzaldehyde only (Figure 3E) and when exposing to dopamine or raclopride (Figure 3F).

The similarity of phenotypes of *R02D3.7(ok1745)* to *cat-1* and *cat-2* mutants was unexpected given that the *R02D3.7(ok1745)* mutant exhibited a high turning frequency and intensified ARS, whereas the dopamine metabolism-deficient mutants did not. Given that *R02D3.7(ok1745)* mutants exhibited an increase in high-angle turning frequency, similar to animals treated with exogenous dopamine (Figure 3B), we anticipated that they would show an opposite phenotype to the dopamine metabolism-deficient mutant phenotype. To further determine whether worms treated with exogenous dopamine also show a deficiency in state-dependence, we performed a state-dependent olfactory adaptation assay using wild-type worms treated with exogenous dopamine or the dopamine antagonist raclopride [2]. Wild-type worms treated with exogenous dopamine showed a deficiency in state-dependence, as seen in the *R02D3.7(ok1745*) mutants (Figure 3F). Similarly, exogenous raclopride treatment also caused a deficiency in state-dependent behavior (Figure 3F). Thus, both *R02D3.7(ok1745)* and the dopamine synthesis-deficient mutant, *cat-2(e1112)*, exhibit defects in state-dependency olfactory adaptation. We conclude that state-dependent olfactory adaptation seems to be an “all-or-none” type of behavior as both effects of dopamine deficiency and dopamine excess appear to impair state-dependency olfactory behavior.

#### 2.3.3. Measurement of Body Size

Dopamine is required to control body size in *C. elegans* [33]. To test whether R02D3.7 affects body size, we measured the body size of *R02D3.7(ok1745)* mutants and found that body size was similar to the body size of N2 wild-type animals (Figure S3). This may be a natural phenomenon, considering the previous finding that there is no change in size when exogenous dopamine is administered to wild-type animals [33]. Interestingly, the double mutant of *R02D3.7(ok1745)* and *cat-2(e1112)* had the same phenotype as the *cat-2(e1112)* single mutant (Figure 3G). Taken together, our findings indicate that the *R02D3.7(ok1745)* mutant mimics the phenotype of worms treated with exogenous dopamine, suggesting increased dopamine signaling in this mutant, which may be caused by dysregulated dopamine release, dopamine production or both. However, it is worth noting that our method of measuring body size could be rendered more precise, for example, by using of microfluidic chip capture under synchronized culture conditions [34].

### 2.4. R02D3.7 Downregulates the Expression of Dopamine Metabolism-Related Genes

**Figure 4:**
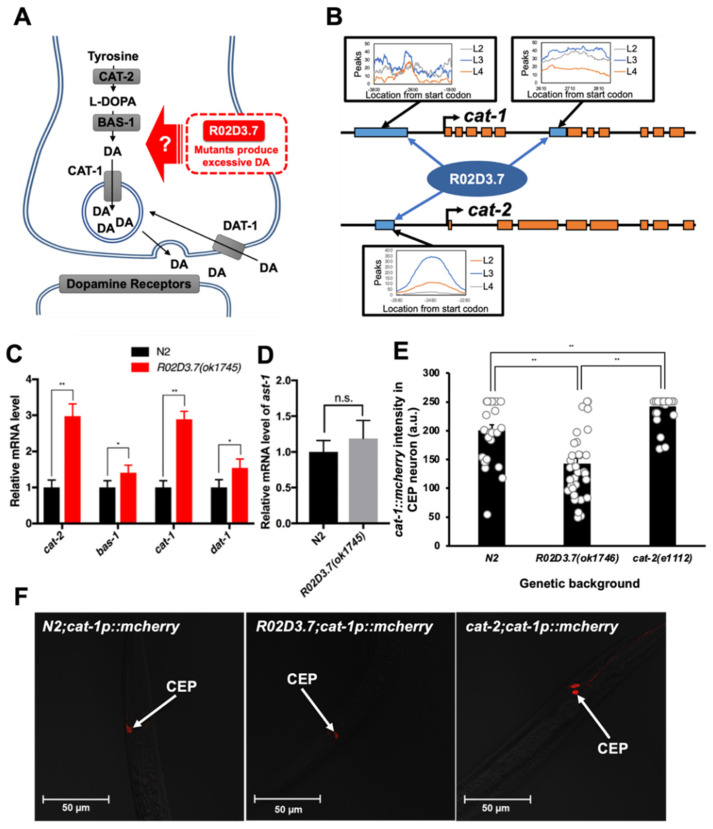
*R02D3.7* regulates dopamine biosynthesis- and transportation-related genes. (**A**) Schematic of predicted R02D3.7-regulated targets in the dopamine biosynthesis and transportation pathways. Abbreviations: BAS-1, biogenic amine synthesis-related-1; DA, dopamine; DAT-1, dopamine transporter-1; L-DOPA, levodopa. (**B**) Illustration of dopamine metabolism-related genes with R02D3.7 binding locations. Orange box indicates *cat-1* and *cat-2* gene exons; blue box indicates the R02D3.7-binding region determined by analyzing ChIP-seq data from the modENCODE database (www.modencode.org, accessed on 30 November 2021) [39,40,41]. The arrows from the blue boxes indicate the binding peak of R02D3.7 at the L2 (orange), L3 (blue), and L4 (grey) stages. (**C**) Dopamine biosynthesis- and release-related gene expression in N2 and *R02D3.7*(*ok1745*) strains. * *p* < 0.05; ** *p* < 0.0001; two-tailed, unpaired Student’s *t*-test. Bars represent the mean ± s.d. of three independent biological replicates. (**D**) Expression of AST-1 in N2 and *R02D3.7*(*ok1745*) strains. n.s., no significant difference (*p* > 0.5); significance was determined using a two-tailed, unpaired Student’s *t*-test. Bars represent the mean ± s.d. of three independent biological replicates. (**E**) The red fluorescence intensity of *cat-1::mCherry* intensity of CEP neuron. Each bar graph was generated by using ZEISS ZEN 3.4 program to analyze the red fluorescence intensities in the CEP neurons in the 8-bit images taken under each transgenic line (values from 0-255). Each measured value (in arbitrary units, a.u.) from the individual worms constituted a rounded circle on each bar graph. Bars are mean ± SEM.; ** *p* < 0.0001; two-tailed, unpaired *t*-test; *n =* 28 for N2;*cat-1p::mCherry*; *n* = 32 for *R02D3.7(ok1745)*;*cat-1p::mCherry,* and *n =* 28 for *cat-2(e1112)*;*cat-1p::mCherry*. (**F**) Confocal images focused in CEP neurons of N2;*cat-1p::mCherry, R02D3.7(ok1745)*;*cat-1p::mCherry,* and *cat-2(e1112)*;*cat-1p::mCherry* transgenic worms. Image gained using 40x magnificent oil-immersion lens on ZEISS LSM900 confocal microscopy Red indicates *cat-1p::mCherry* expression in CEP neuron. Scale bar = 50 μm.

Our results raise the question of the molecular mechanism by which R02D3.7 regulates at least one dopamine metabolism-related gene, because the *R02D3.7(ok1745)* mutant phenotype mimicked the dopamine over-synthesis phenotype (Figure 3 and Figure 4A). There were some genes and derivatives, including the dopamine biosynthetic pathway, identified as candidates for regulation by R02D3.7 (Figure 4A) [35]. CAT-2, which is a homolog of TH, converts tyrosine into L-DOPA [4]. L-DOPA is then converted into dopamine by BAS-1, biogenic amine synthesis-related protein and the *C. elegans* homolog of mammalian aromatic amino acid decarboxylase [36]. CAT-1 is the *C. elegans* homolog of mammalian VMAT2. CAT-1 loads dopamine into synaptic vesicles and facilitates the release of dopamine into synapses [12]. DAT-1 is the *C. elegans* homolog of mammalian dopaminergic transporter and is predicted to mediate the reuptake of synaptic dopamine to control dopamine levels [37,38].

The predicted R02D3.7 structure features two zinc-finger binding motifs, which suggests that R02D3.7 may play a role as a transcription factor [14]. To determine whether the mechanism of the transcriptional regulation of dopamine metabolism-related genes involves the direct action of R02D3.7, we investigated whether R02D3.7, which contains two zinc-finger motifs [14], directly binds to dopamine metabolism-related genes. We searched public transcript databases for *C. elegans* and found that the database maintained by the modENCODE consortium contained chromosome immunoprecipitation-sequencing (ChIP-seq) data to define the binding locations for a large array of transcription factors (www.modencode.org, accessed on 30 November 2021). This database was found to contain information about R02D3.7 binding sites within the *C. elegans* genome [39,40,41]. We reanalyzed the ChIP-seq data and found that R02D3.7 binds to the fifth intron of the *cat-1* gene and to the promoter region of the *cat-2* gene at the third larval (L3) stage (Figure 4B), but not to other dopamine metabolism-related genes, such as *bas-1* and *dat-1* (Figure S3) [35,42].

To confirm that R02D3.7 regulates expression of the *cat-1* gene, we compared the mRNA levels of several genes that are known to regulate dopamine metabolism (e.g., synthesis and release) between wild-type and *R02D3.7(ok1745)* worms at the L3 stage (Figure 4B). Unexpectedly, quantitative transcriptional analysis revealed that there were almost three-fold increases in the mRNA levels of *cat-1* and *cat-2*, and slight increases in the mRNA levels of *bas-1* and *dat-1* (Figure 4C). Thus, R02D3.7 negatively controlled dopamine metabolism-related genes under normal conditions.

Given the increased transcriptional levels of dopamine biosynthetic genes in the *R02D3.7(ok1745)* mutants, we next sought to investigate the common transcriptional regulator axon steering defect (AST-1). The *ast-1* gene is known to encode a transcription factor that activates several dopamine metabolism-related genes, including *cat-1*, *cat-2*, *cat-4*, *bas-1* and *dat-1* [34]. We found no changes in *ast-1* mRNA levels in *R02D3.7(ok1745)* mutants (Figure 4D), suggesting that *ast-1* may not be involved in the R02D3.7-mediated transcriptional regulation of genes involved in dopamine biosynthesis/signaling. Moreover, *rcat-1* expression has been examined under *ast-1(gk463)*. The expression of *rcat-1* did not show any significant difference as to the wild type animals (data not shown). Our data confirmed that R02D3.7 functions within dopaminergic neurons as a negative regulator of *cat-1* and *cat-2* gene expression (Figure 4D), independently of AST-1.

As *cat-1* and *cat-2* genes play key roles in dopamine biosynthesis and secretion, the phenotype observed when these two genes are overexpressed is consistent with the dopamine overproduction phenotype of *R02D3.7(ok1745)* mutants [35]. In addition, the difference in expression levels of *cat-2* and *bas-1* may cause the accumulation of L-DOPA, a precursor of dopamine synthesis that is produced by the actions of the products of these two genes. To test this hypothesis, we measured the intensity of dopaminergic neurons in R02D3.7 mutants, as exogenous L-DOPA has been reported to damage dopaminergic neurons [43,44,45]. We found that the fluorescence intensity of dopaminergic neurons was 30% less in *R02D3.7(ok1745)* mutants than in wild-type animals (Figure 4E, compare left panel vs. middle panel in Figure 4F). In addition, we also found that in *cat-2(e1112)* mutants, which cannot produce L-DOPA, the fluorescence intensity of dopaminergic neurons increased approximately 20% compared to that in wild-type animals (Figure 4E, Compare middle panel vs. right panel in Figure 4F). Thus, we renamed R02D3.7 as regulator of *cat* genes-1 (*rcat-1*). Consequently, the *R02D3.7(ok1745)* mutant was also renamed as *rcat-1(ok1745).* RCAT-1 regulates transcription of dopamine biosynthetic genes, which in turn modulates food searching behavior of *C. elegans*.

## 3. Discussion

**Figure 5:**
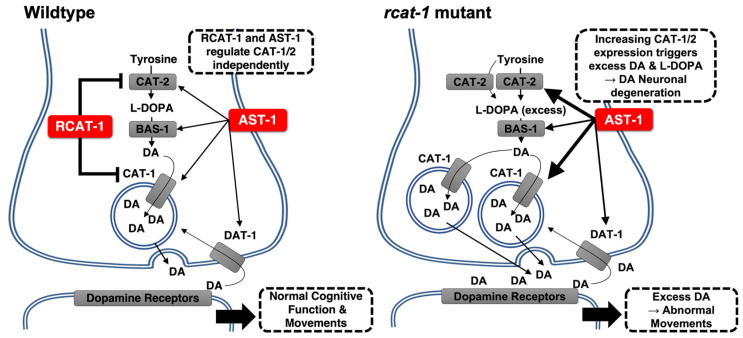
The role of RCAT-1 in dopaminergic neurons of *C. elegans.* RCAT-1 inhibits the transcription of the *cat-1* and *cat-2* genes by direct binding. The mutant *rcat-1(ok1745)* may be deficient in its regulatory function of dopamine metabolism (biosynthesis and release), resulting in aberrant dopamine-modulated behavior.

In this study, we identified RCAT-1 (R02D3.7) as a novel negative transcriptional regulator that suppresses the transcription of *cat-1* and *cat-2* genes, which are essential genes for dopamine metabolism in *C*. *elegans* (Figure 5). The typical phenotype of *rcat-1(ok1745)* mutants showed an increased frequency of high-angled turns and intensified ARS behavior compared with the N2 wild-type animals (Figure 3A,C), indicating the importance of trans-regulatory factors for dopamine release and packaging (*cat-1*/*Vmat2*) and dopamine biosynthesis (*cat-2*/*TH*). We also observed a higher turning frequency, intensified ARS behavior and defects in state-dependent olfactory adaptation in the *rcat-1(ok1745)* mutant (Figure 3A,C and Figure S3). In addition, we found that the number of dopaminergic neurons was reduced in *rcat-1(ok1745)* mutants compared with wild-type animals, such that the mutant showed excessive release of dopamine and the neurons were then damaged by excess L-DOPA (Figure 4E,F). The model of excessive release may be best tested by the direct measurement of dopamine levels at the synapse, which will be performed in a future study.

The phenotype of the mutant *rcat-1(ok1745)*, in which the dopamine metabolism-related genes *cat-1* and *cat-2* were overexpressed due to a lack of transcriptional control, was similar to the previously reported phenotype of increased turning frequency in *C. elegans* exposed to exogenous dopamine [2]. A negative feedback mechanism involving *cat-1*/*Vmat2* usually increases dopamine levels in the cytosol, as the *rcat-1* mutant may cause the uncontrolled release of dopamine into the synapse instead of packaging dopamine into vesicles. Mechanistically, it is plausible that a defect in the binding of RCAT-1 to the predicted regulatory regions of *cat-1* (within the intron and the promoter) and *cat-2* genes (~2 kb upstream; Figure 4B) may cause such dysregulation of dopamine biosynthesis. However, although we were able to obtain binding site information from the modENCODE project [39,40,41], we were not able to define the consensus binding sequence of *rcat-1*. In addition, binding of RCAT-1 to these genes does not necessarily confirm that RCAT-1 regulates the transcription of dopaminergic genes, because there may also be a co-regulator that participates in this process. It should also be noted that *rcat-1* does not rely on AST-1, a common transcriptional regulator for dopamine metabolism-related genes (Figure 4D), for its effects on dopamine biosynthesis and release/packaging. Regarding the dopaminergic neuron-specific expression of *rcat-1*, previous studies of the effects of serotonin on locomotion during food searching [5,46] and our results from serotonin biosynthesis mutants do not support a serotonergic signaling pattern (Figure 2B). In fact, *tph-1(mg280)*, a TH mutant [27], exhibited no change in food-search behavior (Figure 2C). Taken together, our findings indicate that *rcat-1* may be involved in the negative feedback regulation of dopamine metabolism (synthesis and release) in a cell type-specific manner (dopaminergic neurons).

A database search found that there were no mammalian homologs of RCAT-1. However, although this may limit the direct applicability of our findings to inherited neurodegenerative diseases caused by dopamine dysregulation, understanding the general mechanism of RCAT-1 action may suggest new strategies for the regulation of dopamine metabolism and release that include the design of potential therapeutic molecules for DDS (dopamine dysregulation syndrome) that lead to the elucidation of the cause of dopamine-defective neurodegenerative diseases. As the *rcat-1* mutant exhibited excessive release of dopamine into synapses and showed behavioral defects, the identification of biomolecules that ameliorate the phenotype of increased turning frequency and abnormal food-search behavior in the *rcat-1* mutant [2,19] may provide candidate drug targets for the treatment of DDS. As dysfunction of the reward system is also observed in individuals taking dopaminergic medication for a prolonged time [47,48,49], the identification of RCAT-1 in *C. elegans* may facilitate the development of long-awaited therapeutic targets for dopamine-related neurodegenerative diseases.

This animal model of dopamine signaling may be used to address the great challenges presented by many dopamine-related neurological and psychiatric disorders, including PD, schizophrenia and attention deficit hyperactivity disorder [9,50,51]. For example, levodopa (L-DOPA) and dopamine agonists are medications commonly used to treat PD patients with reduced dopamine release resulting from the degeneration of dopaminergic neurons [52,53,54]. However, these medications have side effects, including hypermobility and hallucinations [54,55]. The development of dopamine therapy and other types of medication to reduce such side effects in PD patients is still in the early phase [56,57]. Dopamine dysregulation syndrome (DDS) is a newly identified syndrome in which PD patients exhibit impulse control problems, such as compulsive gambling or shopping, eating disorders and other addictive behavior [58,59]. The common treatment for PD patients with DDS is to restrict the dosage of dopamine or dopamine agonists, which is a strategy that has limitations [48]. Determining the exact mechanisms that underlie the complex dynamics of dopamine and the cause of DDS is of considerable medical importance.

In summary, our findings provide new insights into the transcriptional regulation of dopamine metabolism. We identified a novel transcription factor that likely regulates dopamine metabolism-related genes, and we developed an animal model that mimics the phenotype of PD patients with DDS. Given that there are no known transcriptional suppressors of the *Vmat2* and *TH* genes or their homologs, the discovery of *rcat-1* reinforces the importance of dopamine biosynthesis and release in PD patients and provides a potential therapeutic target [60]. The *rcat-1(ok1745)* mutant may provide a new tool for studying the regulation of VMAT2 and TH in the context of dopaminergic neuron degeneration.

## 4. Materials and Methods

### 4.1. Strains

The following strains used in this study were obtained from the Caenorhabditis Genetics Center: Bristol strain (N2); RB1488 [*R02D3.7(ok1745)*]; RB681 [*cat-1(ok411*)]; CB1112 [*cat-2(e1112)*]; GR1321 [*tph-1(mg280)*]; OH9279 [*cat-1p::mCherry*(*otIs266*)]; RW11460 [*unc-119(tm4063); R02D3.7::H1-wCherry + unc-119(+)*(*stIs11460*)]. The strain YP0106 [*tph-1p::GFP*] was previously generated in our laboratory [25]. The strains [*R02D3.7::GFP; otIs266*], [*R02D3.7(ok1745)*; *otIs266*], [*R02D3.7(ok1745); cat-2(e1112)*], [*cat-2(e1112); otIs2660*], and [*tph-1p::GFP; stIs11460*] were generated using traditional crossing and transformation techniques [61,62]. To generate a transgenic animal containing *R02D3.7::GFP*, a transgene consisting of a 2 kb region upstream of the *R02D3.7* translational start site and the entire *R02D3.7* genomic region were inserted into the vector *pPD95.77*. The resulting construct was then microinjected along with the co-injection marker *rol-6* [62]. Unless otherwise noted, the strains were cultured on solid nematode growth medium (NGM) containing *E. coli* strain OP50 at 20 °C using standard methods. Synchronized cultures were prepared by hypochlorite treatment of gravid adults, as previously described [63].

The following primers were used for genotyping:

*R02D3.7(ok1745)_F*: 5′-GCC AAT TGT GAT TTT TCC AGC A-3′,

*R02D3.7(ok1745)_R*: 5′-CGT TCA GAA CAA TAA ATC TTT GC-3′,

*cat-2 (e1112)_F:* 5′-ACG TGG AAA CTC GGG GCA A-3′ and

*cat-2 (e1112)_R:* 5′-TAC TCC CTC ACA AAA TGT TTA TGT A-3′.

### 4.2. Behavioral Assays

The turning frequency of the worms was calculated based on the distance traveled by a worm being inversely proportional to the turning frequency. The reciprocal of the distance was calculated as: 1/average distance travelled by one turn. Food detection assays were performed in two methods by modifying the chemotaxis assay from previous article [17]. In the first method, one worm was placed on the opposite side of seeded *E. coli* OP50 (O.D. measure: 4.0). The time taken for this worm to reach the seeded *E. coli* OP50 was then determined. The time taken for this worm to reach the seeded *E. coli* OP50 was then determined. For the average calculation, 40–50 worms were examined for each strain. The second method was performed by placing 15–20 worms on the opposite side of seeded *E. coli* OP50, and the number of worms reaching the food within 20 min was measured. Tests were repeated approximately 15–20 times for the average calculation for each strain. Assays for measuring ARS were performed as previously described [2]. Worms were moved from a plate food plate to a transition plate (no food) to remove any residual food attached to the worm. The worm was then transferred to the observation plate (no food) where the turn frequency was recorded. To assess state-dependency olfactory adaptation, we used previously described methods [8]. Odorant was aliquoted onto 5 agar plugs on the lid of adaptation plates (2 µL of 100% benzaldehyde was used). Animals were washed twice in S Basal (0.1 M NaCl, 0.05 M KPO4 [pH 6], 5 mg/L cholesterol [in 100% ethanol]) and once in assay buffer (5 mM KPO4 [pH 6], 1 mM CaCl2, 1 mM MgSO4), then moved on to an adaptation plate, and the plate was sealed. Animals were incubated in all adaptation conditions for 90min, then washed twice with S Basal and once with assay buffer and placed on chemotaxis plates. Chemotaxis was allowed to proceed for 1h, worms were counted. Dopamine and dopamine antagonist treatments were also performed as previously described [2]. Dopamine (0.1, 1.0, or 10 mM) or raclopride (1 mM) was dissolved into the agar (without salts) and allowed to equilibrate overnight at 20 °C [2]. All assays were performed using an SZX7 zoom stereo microscope (Olympus, Tokyo, Japan).

### 4.3. Quantitative Reverse Transcription-Polymerase Chain Reaction

Synchronized populations were collected at the L3 stage, pelleted and frozen at −80 °C. Total RNA, cDNA synthesis and real-time polymerase chain reaction (PCR) were performed as previously described [64]. Total RNA was isolated from samples using RNAspin Mini columns (GE healthcare, Chicago, IL, USA), according to the manufacturer’s instructions. Two micrograms of RNA were reverse transcribed using the Transcriptor first strand cDNA synthesis kit (Roche, Basel, Switzerland) with oligo dT priming. Quantitative PCR was performed using iQ SYBR Green Supermix (Bio-Rad Laboratories, Hercules, CA, USA) according to the manufacturer’s instructions. The reaction products were analyzed using a CFX ConnectTM Real-Time PCR Detection System (Bio-Rad Laboratories, Hercules, CA, USA). Relative mRNA expression levels were determined using the ∆∆CT method, and the mRNA expression level of *act-1* was used as a reference to normalize the results. The sequences of the primers used for quantitative reverse transcription-PCR are listed in Table S1.

### 4.4. DiI Staining Assay

Animals were washed twice with M9 buffer and then transferred to an Eppendorf tube containing a DiI dye solution diluted to 10 mg/mL with M9 buffer. Worms were then incubated for 3 h at 20 °C to stain the sensory cilia, as previously described [65]. Stained animals were placed on an NGM plate seeded with *E. coli* OP50 for destaining.

### 4.5. Confocal Microscopy

To analyze the results of the DiI staining assay and the fluorescence of transgenic lines (e.g., *R02D3.7::GFP*, *R02D3.7::H1-wCherry*, *cat-1p::mCherry* and *tph-1::GFP*), animals were imaged by confocal microscopy. Before imaging, each animal was incubated for 5 min with 50 mM sodium azide in a sample tube. Conditioned animals were placed on 3% agar on a glass slide for viewing under LSM700 and LSM900 confocal microscopes (ZEISS, Jena, Germany) with FITC (to detect *R02D3.7::GFP* and *tph-1::GFP*) and rhodamine (to detect DiI dye, *cat-1p::mCherry* and *R02D3.7::H1-wCherry*) filters. For the quantification of *cat-1p::mCherry* intensity, we used a modification of a previously described protocol [66]. The Z-stack mode was used to acquire cephalic sensillum (CEP) neuron-focused focal plane images, which were separated manually. The intensity of *cat-1p::mCherry* fluorescence was quantified as the maximum values in CEP neurons. All images were 8-bit and were analyzed using ZEN 3.4 software (ZEISS, Jena, Germany).

### 4.6. Body Size Measurement

To optimize the techniques and conditions used for this experiment, we modified previously described protocols [33,66]. To produce adult worms, we placed 10 adult worms in NGM plates containing *E. coli* OP50. After a 2 h egg lay period, the adult worms were removed, and the eggs were incubated for 72 h. After incubation, each adult worm was suspended in M9 buffer, and 50 mM sodium azide was added to prepare the worms for imaging. Anesthetized animals were placed on glass slides containing 3% agar for viewing under the microscope. Images of single worms were captured using an LSM880 confocal microscope (ZEISS, Jena, Germany), and the body size of the worms was determined manually using ImageJ software (National Institutes of Health, Bethesda, MD, USA).

### 4.7. Statistical Analysis

Microsoft Excel (Microsoft, Redmond, WA, USA) and GraphPad Prism 7 (GraphPad Software, San Diego, CA, USA) were used to perform the statistical analysis including graph production. Data presented on the graphs represent the means of more than three independent biological replicates, and the error bars represent the standard deviation or standard error of the mean, as described with in each figure. We used a two-tailed, unpaired Student’s *t*-test and an ordinary one-way analysis of variance, with correction for multiple comparisons, to assess statistical significance. The detailed statistical methods for each experiment are presented in the respective figure legends.

## Data Availability

Not applicable.

## Supplementary Materials

The following supporting information can be downloaded at: https://www.mdpi.com/article/10.3390/ijms23042393/s1.
