# Supplementary material for: Deficiency in RCAT-1 Function Causes Dopamine Metabolism Related Behavioral Disorders in Caenorhabditis elegans
Source: Int J Mol Sci. 2022 Feb 21;23(4):2393. doi: 10.3390/ijms23042393 (PMC8879058; doi:10.3390/ijms23042393)
Supplement: Supplementary file 1 [file ijms-23-02393-s001.zip › Supplement Materials_R2.pdf]

# Supplementary Materials

**Video S1. Movement of N2 wildtype.** The arrow indicates the location of the nematode at the beginning of the video. Video was recorded by an Olympus SZX7 zoom stereo microscope.

**Video S2. Movement of *R02D3.7(ok1745)*.** The arrow indicates the location of the nematode at the beginning of the video. Video was recorded by an Olympus SZX7 zoom stereo microscope.

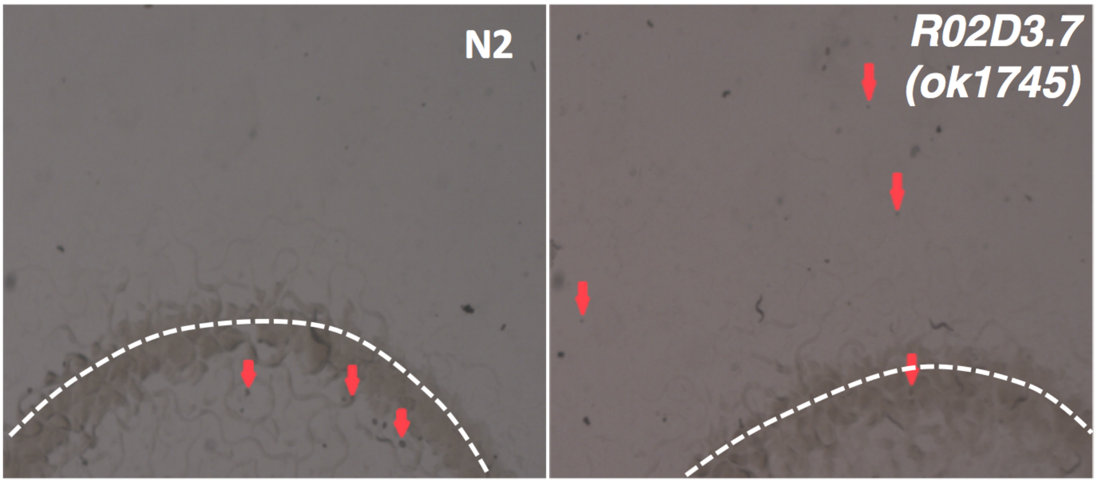


**Figure S1. Egg laying pattern of *R02D3.7(ok1745)*.** *R02D3.7(ok1745)* strain lays egg outside of seeded food unlike the wild-type animals that lay inside of seeded food.

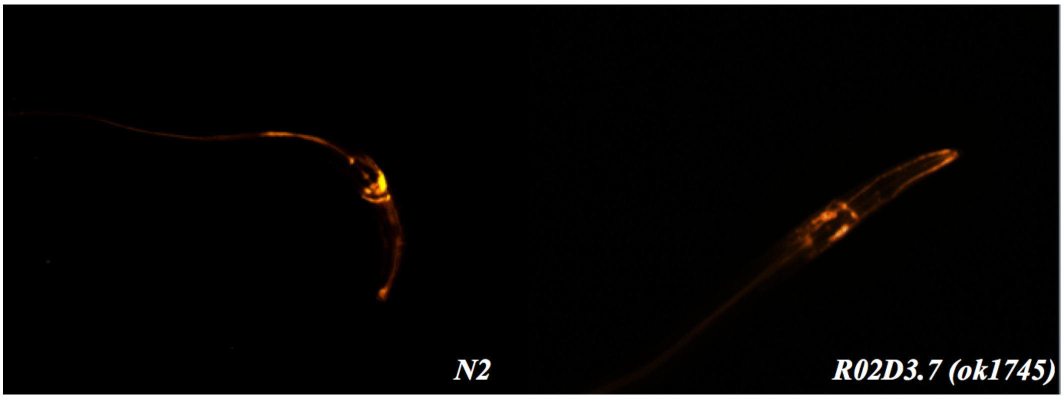


**Figure S2. DiI staining of the wild-type animals and *R02D3.7 (ok1745)* strain.** Staining of chemosensory neurons was performed on both strains. *R02D3.7 (ok1745)* was stained normally as the wild-type N2 animals. This indicates that *R02D3.7 (ok1745)* has no structural defects in chemosensory neurons.

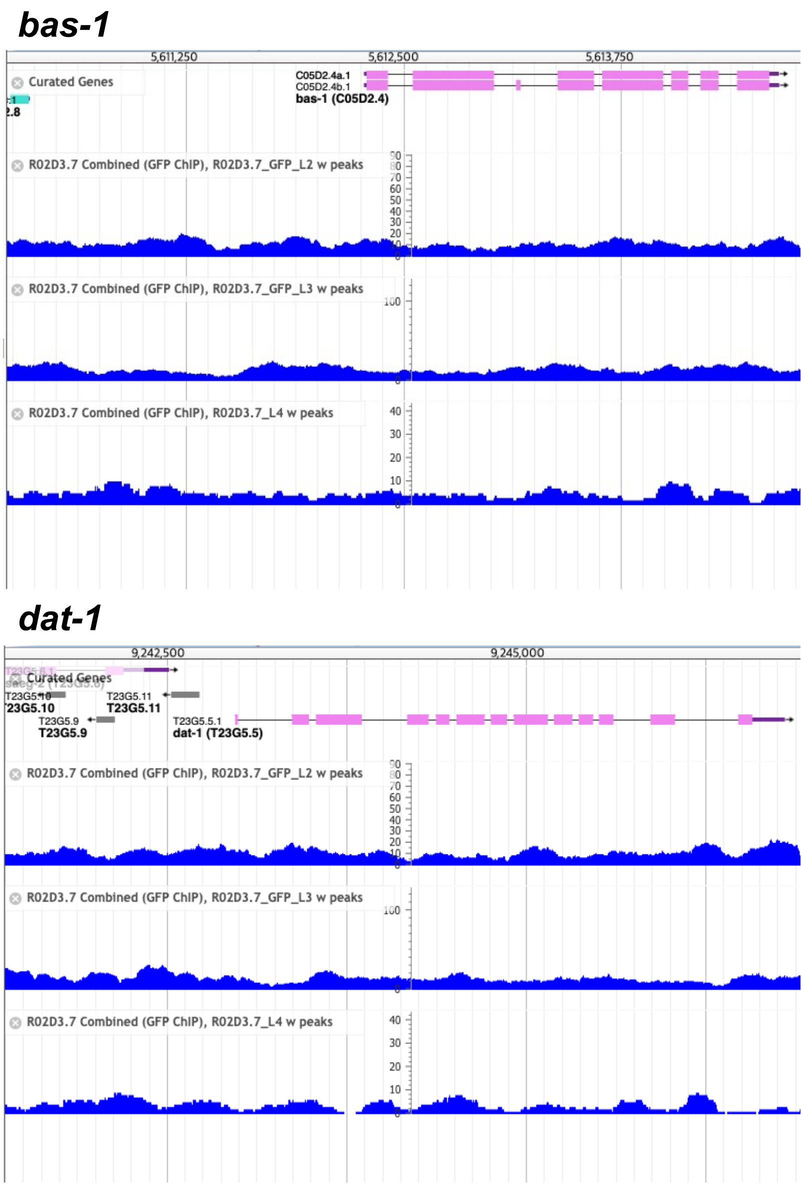


**Figure S3.** ***bas-1*** **and** ***dat-1*** **genes did not bind with R02D3.7.** Illustration is driven from wormbase JBrowere(www.wormbase.org), based on ChIP-seq data analysis from modENCODE database.

**Table S1.** Nucleotide sequence of primers used in qRT-PCR

| Target Gene | Primer Sequence |
|---|---|
| *act-1* | (Forward) GTATGGAGTCCGCCGGA<br>(Reverse) CTTCATGGTTGATGGGGCAA |
| *mec-4* | (Forward) TCAGGGAATGACAGATGAAGTTGC<br>(Reverse) ACATGGGACCTGCTCGAATACTAG |
| *mec-8* | (Forward) GCCAGAAAAATGTTGCAAGGTGTC<br>(Reverse) AATGTTGGAGGCTGGCTGCTG |
| *mec-10* | (Forward) GGAACATGACAGATGAAGTTGCCA<br>(Reverse) CAAGCCATATTGTGGTCCAGCTC |
| *myo-3* | (Forward) CCAGAAGATGGATTCGTCGCC<br>(Reverse) CACAGAAAAGTCCGGAGTATGTGT |
| *unc-22* | (Forward) GTCGTTGAGAACGAAACTGCAATC<br>(Reverse) CGAAGTGACCAACAGTTTGACGT |
| *unc-54* | (Forward) AAGGTTATCGCCAAGCTCGAAC<br>(Reverse) GTTCCTCAGCTTCCTCAACCTG |
| *cat-1* | (Forward) CTTGCTCTCTTCGATGGATCAA<br>(Reverse) ATTTCCGATGGTGATTGCTCCT |
| *cat-2* | (Forward) ATTTGAAAATTCTCCAAGAGG<br>(Reverse) ATGTCTCAAATACGTAGTTGT |
| *bas-1* | (Forward) AACTTTTATGAAATGGGTCTC<br>(Reverse) TCAGAGCAATACATGACAAAT |
| *dat-1* | (Forward) CGTACTCGGCTACATGTCCTGCAA<br>(Reverse) CTGAAAGGCCGGTGATGATAGCTTC |
| *ast-1* | (Forward) AATGATCACTTCACCGGTATGAAC<br>(Reverse) ACGTCACTTTTGCTTTCGGTACTT |
